# Vaccination against influenza among Lebanese health care workers in the era of coronavirus disease 2019

**DOI:** 10.1186/s12889-022-12501-9

**Published:** 2022-01-18

**Authors:** Dalal Youssef, Atika Berry, Janet Youssef, Linda Abou-Abbas

**Affiliations:** 1 Present Address: Ministry of Public Health, Lebanon, Preventive Medicine Department, Ministry of Public Health, Beirut, Lebanon; 2 Institut de santé publique d’épidémiologie et de développement (ISPED), Bordeaux University, France, Bordeaux, France; 3 Al Zahraa hospital University Medical Center, Beirut, Lebanon; 4 Neuroscience Research Center, Faculty of medical sciences, Lebanese University, Beirut, Lebanon

**Keywords:** Health care workers, Influenza vaccination, Coronavirus disease 2019, Knowledge, Attitudes, Practices

## Abstract

**Background:**

Health-care workers (HCWs) are at a higher occupational risk of contracting and transmitting influenza. Annual vaccination is an essential tool to prevent seasonal influenza infection. However, HCWs vaccine hesitancy remains a leading global health threat. This study aims to evaluate the flu vaccination coverage rates among Lebanese HCWs and to assess their knowledge, attitudes, practices, perceived barriers, and benefits toward the flu vaccine during the COVID-19 pandemic. In addition, we sought to identify the factors associated with flu vaccine uptake.

**Methods:**

A cross-sectional study using an online survey was conducted in Lebanon among HCWs between 14 and 28 October 2020. Multivariable logistic regression was carried out to identify the factors associated with influenza vaccine uptake.

**Results:**

A total of 560 HCWs participated in the survey of whom 72.9% were females, and 53.9% were aged between 30-49 years. Regarding Flu vaccination uptake, the rate has risen from 32.1% in 2019-2020 to 80.2% in 2020-2021 flu season. The majority of HCWs had a good knowledge level and a positive attitude toward flu vaccination. Regarding their practices, less than 50% of HCW were currently promoting the importance of getting the flu vaccine. The majority (83.3%) ranked the availability of a sufficient quantity of vaccines as the most significant barrier to flu vaccination. The main perceived flu vaccination benefits were enhancing patient safety, minimizing the viral reservoir in the population, decreasing hospital admission, and avoiding influenza and COVID-19 co-infection. The odds of influenza vaccine uptake was lower in unmarried compared to married HCWs (OR = 0.527, CI (0.284-0.978). However, HCWs having received the influenza vaccine in the previous season (OR = 6.812, CI (3.045-15.239)), those with good knowledge level (OR = 3.305, CI (1.155-9.457)), low perceived barriers (OR = 4.130, CI (1.827-9.334)) and high perceived level of the benefits (OR = 6.264, CI (2.919-13.442)) of the flu vaccination were found more prone to get the flu vaccine.

**Conclusion:**

Flu vaccination uptake has increased among HCWs during the 2020-2021 flu season compared with the previous one. Continuing education as well as ensuring free, equitable, and convenient access to vaccination are still required to increase the annual flu vaccination uptake among HCWs.

## Introduction

Influenza is a highly contagious respiratory disease that poses a major global public health threat [1]. This debilitating illness spreads mainly by droplets and leads to substantial morbidity and fatality annually [2]. There are four influenza viruses types: A, B, C, and D. In particular, types A and B can cause seasonal epidemics in humans specifically influenza A [1, 2]. Cases infected by influenza are often mild and characterized by fever associated with respiratory as well as systemic symptoms such as muscle or body aches, headache, and fatigue. However, the course of influenza can be severe, since some cases can require hospitalization and admission to the intensive care unit. The severity of influenza among cases in any particular year is determined by a variety of factors and conditions, including age and comorbidities, and reflects the degree of genetic drift or shift in the dominant influenza virus strain, as well as vaccine efficacy and coverage [3]. Higher morbidity and mortality rates are noticed particularly in high-risk groups including infants, elderly people, pregnant women, individuals with underlying medical conditions, and health care workers (HCWs) [4].

Keeping in mind that the world will confront a greater problem this year: Seasonal influenza, which is still not widely preventable, will be confounded by the severe acute respiratory syndrome coronavirus 2 (SARS-CoV-2) that leads to coronavirus disease 2019 (COVID-19) [5]. Influenza strains are expected to circulate alongside SARS-COV-2, and it is widely established that the two viruses can infect the same patient [6]. The two viruses are considerably different pathogens, but they have overlapping signs and symptoms.

Health-care workers (HCWs) are not only at a higher occupational risk of contracting influenza and COVID-19 viruses, but they are also a significant source of virus transmission to their colleagues, families, and patients as well as virus circulation in the community [7]. HCWs in practice often use non-pharmacological interventions (NPI) as protective measures during the influenza season [8]. Flu vaccination is an essential tool for reducing outbreaks and disease severity, particularly in high-risk groups. Influenza vaccines are known to be 60% protective among healthy people and decrease illness duration and severity in symptomatic individuals [9]. Thus, health institutions such as the World Health Organization (WHO) and the Strategic Advisory Group of Experts (SAGE) on immunization recommended the prioritization of HCWs vaccination against seasonal influenza [10, 11]. However, vaccine hesitancy among HCWs remains a leading global health threat and vaccination coverage among HCWs continues to remain low over the world. The lack of influenza vaccine availability, as well as misconceptions about vaccine safety and effectiveness are all factors contributing to low vaccine uptake [12].

In Lebanon, the ministry of public health issued a memorandum No. 149 on October 6th, 2020 recommending annual flu vaccination for HCWs and identifying the target categories that must be targeted for immunization. However, the influenza vaccine is not included in the national immunization program; as a result, it is neither mandated nor financed by the Ministry of Public Health. Thus, out-of-pocket vaccine expenses are paid by households. In addition, no national data on annual influenza vaccine coverage are published in Lebanon. A cross-sectional survey performed in 30 pharmacies randomly selected across Lebanon revealed an overall vaccination rate of 27.6%. This coverage might be an overestimation of the actual prevalence of vaccination in the Lebanese adult population since the subjects were interviewed in community pharmacies [13]. Concerning knowledge, attitude, and practice among Lebanese HCWs regarding influenza vaccines, there is a lack of information. Therefore, the objectives of the present study were to evaluate the flu vaccination coverage rates and to assess HCW’s knowledge, attitudes, and practices (KAP) toward the seasonal influenza vaccine during the COVID-19 pandemic. In addition, we sought to identify the factors associated with influenza vaccine uptake. Results of the present study could help health policymakers in implementing effective interventions that will increase influenza vaccine uptake among HCWs.

## Methods

### Study design and participants

A cross-sectional study, using an online survey, was conducted between 14 and 28 October 2020 before the onset of the influenza seasonal outbreak in Lebanon. All HWCs, working in Lebanese hospitals in different provinces in Lebanon and who agreed to participate in the study, were eligible for participation. As the Lebanese government recommended the public to minimize face-to-face interaction, potential respondents were electronically invited to participate.

### Instrumentation

An extensive review of the literature was conducted to identify relevant items on flu vaccination knowledge, attitude, and preventive practices among HCWs. The authors developed a structured questionnaire which was then reviewed by a panel of 4 experts that included a medical doctor, epidemiologist, infectious diseases expert, and a hygienist. They were asked to evaluate its content validity based on the relevance, coverage, and representativeness of the items in assessing the HCWs knowledge, attitude, and practice toward flu vaccination. The content validity index (CVI) was calculated for each item. Of the total items, three were rated irrelevant, thus, they were omitted from the questionnaire which was translated and adapted to the Arabic language. Then, a pilot study was performed on 10 HCWs to evaluate the comprehensibility and clarity of the items. Minor linguistic edits were made.

The survey was developed and was divided into five sections:

1. **Socio-demographic characteristics:** This section includes age, gender, marital status, urbanicity, specialty, place of work, type of hospital, clinical experience, health status, underlying conditions, and health coverage. Participants were also asked whether they have received the influenza vaccine in the past season and if they have previously refused any kind of vaccination.
2. **Knowledge section:** Six domains were designed to assess HCWs knowledge toward flu vaccination: Nature, symptoms, and transmission of influenza (6 items), overlapping between COVID-19 and influenza (3 items), vaccine effectiveness and safety (5 items), administration, storage and handling (3 items), target groups for vaccination (4 questions) and vaccination Timing (3 items). All the items, except item K15, were answered on a true/false and “do not know” option. A correct response was given a value of ‘1′ and a “wrong” or “don’t know” response was assigned a value of ‘0′. Item K15 exploring the prioritized groups for flu vaccination was in the form of a multiple-choice question in which a value of 1 was assigned for correct answers and 0 for incorrect or unknown answers. Hence, an overall knowledge score would range from 0 to 28 points. HCWs were categorized as having good knowledge if the score was between 80 and 100% (22–28 points), moderate if the score was between 50 and 79% (14–21 points), and poor if the score was less than 50% (< 14 points).
3. **Attitudes toward influenza vaccine:** Two dimensions with a total of 15 items were used to assess HCW attitudes toward influenza and vaccination (9 items) and health facility, government, and society (6 items). Responses to questions related to attitude were graded on a 5-point Likert scale, an agreement scale ranging from ‘1’ for strongly disagree to ‘5’ strongly agree. A point of 1 was given to the 2 options “strongly agree” and “agree” answers while strongly disagree, disagree or neutral responses were given a 0 point. The overall level of attitude was categorized using original Bloom’s cut-off point, as positive if the score was 80–100% (12–15 points), neutral if the score was 50–79% (8–11 points) and negative if the score was less than 50% (< 8 points).
4. **Practices toward flu vaccination**: Two main domains constitute this section. The first one was about the uptake of flu vaccination and it was based on only one question regarding willingness to vaccinate during the current influenza season. The item was answered “yes” and “no”. The answer “yes” was assigned 1 point whereas the “no” answer was assigned 0 points. The second domain was about practices and behaviors related to vaccine promotion and continuous education (training). Thirteen questions were used to evaluate positive behaviors.
5. **Perceived barriers and benefits:** this section comprises 7 items concerning the main barriers and 5 items reflecting the flu vaccination benefits as perceived by HCWs.

The survey was pilot-tested on 5% of the sample to check the clarity and readability of all items and to evaluate the time needed to complete the survey. HCWs did not report any problems in understanding the survey. The average time for completing the survey was 8 min.

### Sample size calculation

The Raosoft sample size calculator designed specifically for population surveys was used to calculate the required sample size. Assuming that 50,000 registered HCWs are actively practicing at the health facilities level, a 95% confidence level, and an absolute error of 5%, the minimum required sample size was 381.

### Data collection

Directors of government-run and private hospitals were contacted via email and asked to participate in the study. They were also asked to designate a focal person who would be responsible for sharing the survey with all HCWs at their facilities. Following their consent, an online questionnaire was emailed to the designated focal person via “WhatsApp”.

An informed consent form, including the research purpose, information confidentiality, and right to withdraw the participation at any time without prior justification, was attached to the online survey. Respondents were asked to confirm their willingness to participate in the study by answering a yes-no question. After confirmation of the question, HCWs were directed to complete the online survey. All the necessary measures to safeguard participants’ anonymity and confidentiality of information were respected.

### Data analysis

All the statistical analyses were performed using the statistical software SPSS (Statistical Package for Social Sciences), version 22.0. Categorical variables were reported by frequencies and percentages. Chi-square test was used to determine the associations between influenza uptake and independent variables including demographic characteristics, perceived barriers, and benefits as well as KAP scores. Multivariable logistic regression was also carried on the significant variables in the bivariate analyses/chi-squared test with a *p*-value <0.2 to identify the factors associated with influenza uptake among HCWs. For all tests, *P* values <0.05 were considered statistically significant.

## Results

### Baseline characteristics of the study participants

Table 1 shows the baseline characteristics of the participants. A total of 560 HCWs participated in the survey of whom 72.9% were females. About two-third were married (66.1%) and approximately half of them (53.9%) were aged between 30-49 years. The majority of them were nurses (63.2%). Of the total, 75.4% of participating HCWs are working mainly in private settings. Regarding their work experience, 43.8% of the participants had a large work experience (more than 10 years of experience).

**Table 1:** Socio-demographic characteristics of the participants (N = 560)

	***n***	%
**Gender**		
Male	152	27.1%
Female	408	72.9%
**Age (years)**		
20-29	219	39.1%
30-49	302	53.9%
> 50	39	7.0%
**Marital status**		
Married	370	66.1%
Unmarried	190	33.9%
**Urbanicity**		
Urban	314	56.1%
Rural	246	43.9%
**Type of hospital**		
Private	422	75.4%
Public	138	24.6%
**Occupation**		
Physician	81	14.5%
Lab specialist	74	13.2%
Pharmacist	51	9.1%
Nurse	354	63.2%
**Years of experience**		
Less than 10 years	315	56.3%
10 years or more	245	43.8%
**Heath status**		
Fair or below	86	15.4%
Good or above	474	84.6%
**Presence of comorbidities**		
Yes	135	24.1%
No	425	75.9%
**Type of hospital**		
Private	422	75.4%
Public	138	24.6%
**Health Coverage**		
Public insurance	490	87.5%
Private insurance	29	5.2%
None	41	7.3%
**Influenza vaccine uptake in previous season**		
Yes	180	32.1%
No	380	67.9%
**Refused vaccination of a certain type of vaccine in the past**		
Yes	82	14.6%
No	478	85.4%

N frequency, % percentage

### Flu vaccination uptake

Of the total HCWs, only one-third (32.1%) declared their uptake of the influenza vaccine the last year. However, the majority of HCWs (80.2%) declared their willingness to uptake the influenza vaccine the current year.

### HCWs knowledge toward flu vaccination

Of the total HCWs (77%) had a good level of knowledge. Table 2 illustrates the knowledge about vaccination against novel Coronavirus among the HCWs. The majority of the respondents were aware of the nature, symptoms and, transmission of influenza (70%), the importance and the safety of the influenza vaccine (77%) as well as the timing of vaccination (71.5%). Knowledge was also good for the overlapping of two respiratory viruses (COVID-19 and influenza) domain (70%). Poor knowledge was more apparent in response to questions related to the awareness regarding the influenza vaccine target groups (30%). Table 3 describes HCWs answers toward influenza vaccine knowledge items. Despite the good level of knowledge recorded in the domain related to the nature and transmission of influenza, almost 49.5% of HCWs were not knowledgeable that symptoms appear 8 to 10 after exposure to the influenza virus. In addition, 23.8% of them didn’t recognize that the influenza vaccine can continue to be offered as long as influenza viruses are circulating. The unveiled poor knowledge in the vaccine target groups domain shown in Table 2 was particularly related to the question concerning the prioritization of target groups when a limited supply of vaccine is experienced, where more than 60% of HCWs didn’t consider children aged 6 months through 4 years (59 months) and pregnant woman as a priority for immunization in such situation.

**Table 2:** HCWs Knowledge levels towards the seasonal influenza vaccine (N = 560)

	Poor (<50%)	Moderate (50-79%)	Good (≥80%)
	***n*** (%)	***n*** (%)	***n*** (%)
**Domains of Knowledge score**			
Domain1: Influenza: nature, symptoms and transmission	52(9.2%)	114(20.4%)	394(70.4%)
Domain 2: Influenza vaccine importance and safety	39(7%)	90(16%)	431(77%)
Domain 3: Influenza vaccine target groups	**168(30%)**	107(19.1%)	285(50.9%)
Domain 4: Vaccine administration and storage	74(13.2%)	170(30.4%)	316(56.4%)
Domain 5: Timing of vaccination	22(3.9%)	138(24.6%)	400(71.5%)
Domain 6: Influenza and COVID-19	18(3.2%)	150(26.8%)	392(70%)
**Knowledge score (28 items)**	**2(0.4%)**	**290(51.8%)**	**268(47.9%)**

N frequency, % percent

**Table 3:** HCWs answers regarding their Knowledge about influenza vaccination (N = 560)

	#	Correct	Wrong
		***n***(%)	***n***(%)
**Influenza**: **nature, symptoms and transmission (n = 6 items)**			
Influenza, caused by a virus can be a serious disease that can lead to hospitalization and sometimes even death	1	498(88.9%)	62(11.1%)
Anyone can’t get very sick from flu including people who are healthy	2	451(80.5%)	109(19.5%)
The signs and symptoms of influenza include fever, headache, sore throat, pain and aches	3	554(98.9%)	6(1.1%)
You can get flu from patients and coworkers who are sick with flu	4	558(99.6%)	2(0.4%)
If you become sick with flu, you can spread it to others even if you don’t feel sick	5	422(75.4%)	138(24.6%)
Symptoms typically appear 8 to 10 days after a person is exposed to influenza	6	283(50.5%)	277(49.5%)
**Influenza vaccine safety and importance (n = 5 items)**			
The seasonal vaccine protects against the most common influenza viruses including H1N1	1	488(87.1%)	72(12.9%)
Flu vaccine cannot cause flu	2	361(64.5%)	199(35.5%)
Flu vaccines are safe, serious problems from a flu vaccine are very rare	3	497(88.8%)	63(11.2%)
MOPH recommends that HCWs receive influenza vaccine	4	518(92.5%)	42(7.5%)
By getting vaccinated, you help protect yourself, your family, and your patients.	5	517(92.3%)	43(7.7%)
**Influenza vaccine target groups (n = 4 items2)**			
Vaccination to prevent flu is particularly important for people who are at high risk of developing serious flu complications	1	540(96.4%)	20(3.6%)
Pregnant women and people with certain chronic health conditions can get a flu shot	2	382(68.2%)	178(31.8%)
Children younger than 6 months of age are too young to get a flu should not get flu shot	3	409(73%)	151(27%)
When vaccine supply is limited, vaccination efforts should be prioritized to:	4		
Children aged 6 months through 4 years (59 months);	4a	224(40%)	336(60%)
People with chronic diseases pulmonary, cardiovascular, renal, hepatic, neurologic	4b	463(82.7%)	97(17.3%)
People who are immunosuppressed	4c	408(72.9%)	152(27.1%)
Pregnant woman	4d	199(35.5%)	361(64.5%)
Health care personnel;	4e	430(76.8%)	130(23.2%)
**Administration and storage of vaccine (n = 3 items)**			
A trivalent flu shot made using an adjuvant is approved for administration for people ≥65 y of age and older.	1	372(66.4%)	188(33.6%)
Antibodies develop in the body about 2 weeks after influenza vaccination.	2	465(83.0%)	95(17%)
Influenza vaccine should be stored at 2 to 8 ° C	3	517(92.3%)	43(7.7%)
**Timing of vaccination (n = 3 items)**			
Vaccination should occur before onset of influenza activity in the community.	1	545(97.3%)	15(2.7%)
Vaccination should continue to be offered as long as influenza viruses are circulating	2	427(76.3%)	133(23.8%)
Annual vaccination is needed to get the “optimal” or best protection against flu	3	523(93.4%)	37(6.6%)
**COVID-19 and influenza (n = 3 items)**			
Flu vaccine didn’t protect against COVID-19	1	481(85.9%)	79(14.1%)
Both viruses influenza and COVID-19 are transmitted by respiratory droplets	2	554(98.9%)	6(1.1%)
COVID-19, and influenza are vastly different pathogens, but there are important areas of overlap	3	459(82%)	101(18%)

N frequency, % percentage

### HCWs attitudes toward flu vaccination

Out of the 560 HCWs, the majority 471 (84.1%) had positive attitude toward flu vaccination. Table 4 describes HCW’s attitude toward flu vaccination. The majority disagree about considering the influenza vaccine as not compulsory (75.1%) and about considering influenza as a mild disease not necessitating vaccination against (80.9%). Almost half (53.6%) of the participants disagree that disease avoidance benefits are not enough and that healthy people do not need vaccination (61.2.%) while only 9.1% of HCWs agreed that vaccines weaken or overload the immune system and that allergies are on the rise due to the vaccination (12.8%). Of the total HCWs, only 22.8% agreed that is better to develop natural immunity rather than getting vaccinated. It is worth noting that although that 64.2% of them consider vaccines among the safest and most tested medicinal products only 40% agreed that the adverse reactions resulting from flu vaccination were underestimated.

**Table 4:** HCWs’ attitudes toward influenza vaccination items (*N* = 560)

	Strongly disagree	Disagree	Neutral	Agree	Strongly agree
	***n***(%)	***n***(%)	***n***(%)	***n***(%)	***n***(%)
**General attitudes towards influenza and vaccination (*N*** **= 560)**					
I think it is not compulsory for HCW to get vaccinated for Influenza	241(43%)	174(31.1%)	55(9.8%)	70(12.5%)	20(3.6%)
I think that influenza is not a serious condition and therefore is not worth vaccination against.	192(34.3%)	261(46.6%)	53(9.5%)	50(8.9%)	4(0.7%)
I think that the benefits of avoiding the disease are not enough	86(15.4%)	214(38.2%)	114(20.4%)	121(21.6%)	25(4.5%)
I think vaccines weaken or overload the immune system	141(25.2%)	298(53.2%)	70(12.5%)	43(7.7%)	8(1.4%)
It is better for me to develop natural immunity by getting sick rather than to get a vaccine	74(13.2%)	240(42.9%)	118(21.1%)	111(19.8%)	17(3%)
I think healthy people do not need to be vaccinated	74(13.2%)	269(48%)	90(16.1%)	115(20.5%)	12(2.1%)
I consider that allergies are on the rise due to vaccinations	69(12.3%)	282(50.4%)	137(24.5%)	69(12.3%)	3(0.5%)
I think that frequency of adverse reactions to influenza vaccines is underestimated	17(3%)	122(21.8%)	197(35.2%)	206(36.8%)	18(3.2%)
Vaccines are among the safest and most tested medicinal products	10(1.8%)	46(8.2%)	145(25.9%)	324(57.9%)	35(6.3%)
**HCWs Attitudes towards health facility, government and society (*N*** **= 560)**					
I think that health care facilities should ensure availability of influenza vaccine at their institutions	54(9.6%)	136(24.3%)	122(21.8%)	217(38.8%)	31(5.5%)
I think that the government should finance the vaccine for all	7(1.3%)	27(4.8%)	47(8.4%)	202(36.1%)	277(49.5%)
I think Lebanese society has more important problems than influenza	42(7.5%)	99(17.7%)	92(16.4%)	195(34.8%)	132(23.6%)
I think vaccine policy in Lebanon is influenced by financial profits of pharmaceutical companies	9(1.6%)	56(10%)	141(25.2%)	217(38.8%)	137(24.5%)
I think that vaccine information provided by health authorities and scientific societies is reliable	15(2.7%)	66(11.8%)	185(33%)	268(47.9%)	26(4.6%)

N frequency, % percentage

Out of 560, 327(58.4%) consider that Lebanese society has more challenges than influenza. The majority of them (85.6%) declared that the government should finance the vaccine for all and that vaccine policy in Lebanon is influenced by the financial profits of pharmaceutical companies (63.3%). More than 40% of HCWs consider that the health facility where they work should ensure the availability of influenza vaccines at their institution. Whereas, approximately half of the participants (52.5%) considered that information provided by health authorities is reliable and 33% of them were neutral regarding this issue.

### HCWs practices toward flu vaccination

Table 5 summarizes the positive behaviors reported by HCWs regarding vaccine promotion and training. More than 50% of HCWs declared that they continuously encourage their patients, colleagues, and family members to get vaccinated. About 40.5% of HCWs always communicate the importance of vaccination during hospital and clinic visits and 43.4% of them do it occasionally. However, the use of phone and email as tools to promote the importance of flu vaccination was less reported among HCWs. Nearly half of HCWs declared that they never use this option (mobile, email …) or celebrate events related to flu vaccination (vaccine day). Similarly, the majority of HCWs (53.2%) didn’t send vaccine reminders to patients. However, more than 70% of HCWs use printing materials like brochures and posters continuously (23.8%) or occasionally (47.1%) to elucidate the importance of vaccination. The majority of HCWs (91.8%) have attended training about vaccination in the past and only 8.2% have never joined any training focusing on influenza. Of the total, 46.3% of HCWs always encourage other staff and colleagues to attend such conferences and training.

**Table 5:** HCWs Practices toward influenza vaccination

	Never	Occasionally	Always
	*n*(%)	*n*(%)	*n*(%)
**Vaccine promotion and Advertising**			
I encourage my patients to get flu vaccine	36(6.4%)	213(38%)	311(55.5%)
I encourage my colleagues and the office staff to get flu vaccine	46(8.2%)	194(34.6%)	320(57.1%)
I encourage my family members who need to be vaccinated to get vaccinated	43(7.7%)	182(32.5%)	335(59.8%)
I encourage HCWs to get flu vaccine to minimize sick days, loss of productivity and to ensure patient safety	46(8.2%)	167(29.8%)	347(62%)
I encourage HCWs to get flu vaccine to avoid dual infection by COVID-19	41(7.3%)	177(31.6%)	342(61.1%)
I encourage HCWs to get vaccinated to set an example to other workers	62(11.1%)	156(27.9%)	342(61.1%)
**Addressing vaccine hesitance**			
I communicate the importance of getting influenza vaccine during office/clinics visits	90(16.1%)	243(43.4%)	227(40.5%)
I communicate the importance of getting influenza vaccine by telephone or by email	268(47.9%)	193(34.5%)	99(17.7%)
I use brochures and posters in my clinic/office revealing the importance of vaccine	163(29.1%)	264(47.1%)	133(23.8%)
I send influenza vaccine reminder by text to my patient	298(53.2%)	165(29.5%)	97(17.3%)
I celebrate event related to vaccination (vaccination day…..)	285(50.9%)	184(32.9%)	91(16.3%)
**Trainings and Workshops**			
I participate in trainings related to influenza vaccine in the past	46(8.2%)	147(26.3%)	367(65.5%)
I encourage my staff (HCWs) to participate in trainings related to influenza vaccine	74(13.2%)	227(40.5%)	259(46.3%)

N frequency, % percentage

### Perceived barriers and benefits toward flu vaccination

**Fig. 1:**
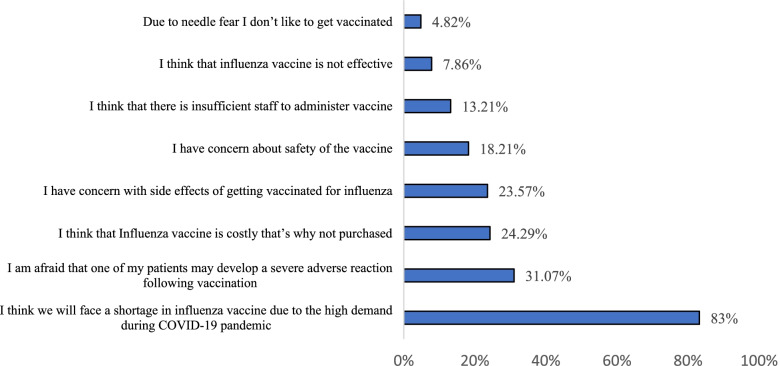
Perceived influenza vaccination barriers by HCWs (N = 560)

Figure 1 presents the perceived barriers to flu vaccination. The majority of our HCWs (83.3%) perceived the availability of a sufficient quantity of vaccine as a barrier to flu vaccination. The fear of a severe adverse event development was stated as a barrier by 31.07% of HCWS followed by the cost (24.29%) and the concern about side effects resulting from vaccination (23.57%). Approximately 18.21% of HCWs were concerned about influenza vaccine safety. A slight proportion of HCWs (7.86%) assumed that the influenza vaccine is not effective and only 4.86% of them showed concern due to the fear from the needle.

**Fig. 2:**
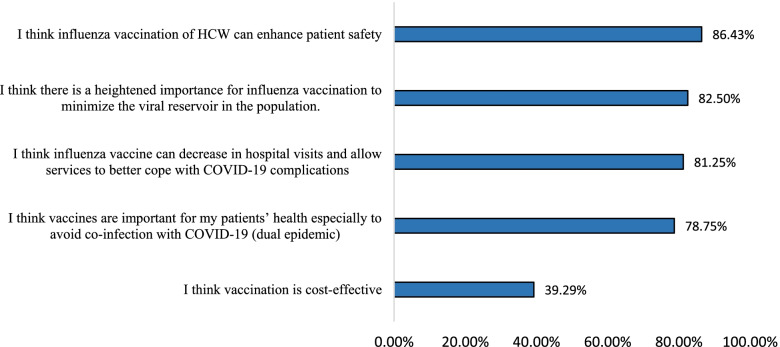
Perceived influenza vaccination benefits by HCWs (N = 560)

Figure 2 shows the benefits of flu vaccination perceived by HCWs. The majority of HCWs thought that their flu vaccination can enhance patient safety (86.43%). In addition, they believed that it can minimize the viral reservoir in the population (82.5%), decrease hospital visits (81.25%) and avoid co-infection by influenza and COVID-19 (78.75%) allowing consequently health services to better cope with COVID-19 complications. Only 39.29% of HCWs consider flu vaccination cost-effective.

### Factors associated with influenza uptake

Table 6 represents the multivariable logistic regression of the factors associated with influenza uptake. Our results showed that the the vaccination uptake was lower in single and divorced HCWs than their counterparts who were married (adjusted OR = 0.527 with 95% CI 0.284 to 0.978). The odds of influenza uptake practice was 6.812 times higher among HCWs who have received the influenza vaccine in the past season compared to those who were not vaccinated in the last season (Adjusted OR = 6.812 with 95% CI of 3.045 to 15.239). Participants with good knowledge of the vaccine’s safety were 3.305 times more conceivable to get the vaccine (OR = 3.305, with 95% CI 1.155 to 9.457) than other HCWs with poor knowledge. With regards to perceived benefit, HCWs who had high and moderate perception levels of benefits were more likely to get vaccinated than their counterparts with a low perception of benefits (OR = 6.264 with 95% CI 2.919 to 13.442). Furthermore, the influenza vaccine uptake was 4.130 higher among HCWs who had the perception of a low barrier compared to those with a high perception of barriers (OR = 4.103 with 95% CI 1.827-9.334).

**Table 6:** Factors associated with influenza vaccine uptake (N = 560)

	Influenza vaccine uptake					
	No	Yes	***p***-value	ORa	95% CI	
	***n*** = 111(%)	***n*** = 449(%)			Lower	Upper
**Gender**			0.612			
Male	28(18.4%)	124(81.6%)				
Female	83(20.3%)	325(79.7%)				
**Age (years)**			0.604			
< 30	41(18.7%)	178(81.3%)				
30-49	64 (21.2%)	238(78.8%)				
50 and above	6(15.4%)	33(84.6%)				
**Urbanicity**			0.489			
Urban	59(18.8%)	255(81.2%)				
Rural	52(21.1%)	194(78.9%)				
**Occupation**			0.273			
Physician	12(14.8%)	69(85.2%)				
Pharmacist	12(16.2%)	62(83.8%)				
Lab technician	14(27.5%)	37(72.5%)				
Nurses	73(20.6%)	281(79.4%)				
**Marital status**			0.041			
Married	71(19.2%)	299(80.8%)		1.00		
Unmarried	40(21.1%)	150(78.9%)		0.527	0.284	0.978
**Perceived Health status**			0.370			
Good and Above	97(20.5%)	377(79.5%)				
Fair and below	14(16.3%)	72(83.7%)				
**Presence of comorbidities**			0.851			
Yes	26(19.3%)	109(80.7%)				
No	85(20%)	340(80%)				
**Type of the hospital**			0.118			
Private	90(21.3%)	332(78.7%)				
Public	21(15.2%)	117(84.8%)				
**Health Coverage**			0.605			
Public	94(19..2%)	396(80.8%)				
Private	7(24.1%)	22(75.9%)				
None	10(24.4%)	31(75.9%)				
**Years of experience**			0.926			
< 10 years	62(19.7%)	253(80.3%)				
> 10 years	49(20%)	196(80%)				
**Influenza vaccine uptake in previous season**			<0.001			
No	100(9.9%)	280(37.6%)		1.00		
Yes	11(6.1%)	169(93.9%)		6.812	3.045	15.239
**Knowledge toward influenza vaccination**			<0.001			
Poor Knowledge	26(66.7%)	13(33.3%)		1.00		
Moderate Knowledge	28(31.1%)	62(68.9%)		2.205	0.703	6.913
Good Knowledge	57(13.2%)	374(86.8%)		3.305	1.155	9.457
**Attitude toward influenza vaccination**			0.065			
Negative	30(60%)	20(40%)				
Neutral	22(56.4%)	17(43.6%)				
Positive	59(12.5%)	412(87.5%)				
**Perceived benefits**			<0.001			
Low	37(56.1%)	29(43.9%)		1.00		
Moderate	27(29%)	66(71%)		3.433	1.466	8.040
High	47(11.7%)	354(88.3%)		6.264	2.919	13.442
**Perceived barriers**			<0.001			
High	29(53.7%)	25(46.3%)		1.00		
Moderate	36(31.3%)	79(68.7%)		1.808	0.783	4.177
Low	46(11.8%)	345(88.2%)		4.130	1.827	9.334

† others included single, widowed, and divorced, N frequency, % percentage, ORa adjusted odds Ratio, 95%CI 95% Confidence interval, *p*-value<0.05 is considered significant

## Discussion

To the best of our knowledge, this study is the first in Lebanon to evaluate the flu vaccination uptake rate among hospital-based HCWs during the COVID-19 pandemic and to explore knowledge, attitudes, practices, and perceived barriers and benefits toward flu vaccination. In addition, we sought to identify the factors associated with flu vaccination uptake during the ongoing COVID-19 pandemic. Results of the present study revealed that flu vaccination uptake has risen from 32.1% in 2019-2020 to 80.2% in 2020-2021. The majority of Lebanese HCWs have a good knowledge level and a positive attitude toward flu vaccination. Of the total, (83.3%) of HCWs ranked the availability of a sufficient quantity of vaccine as the most significant barrier to flu vaccination. The main perceived flu vaccination benefits were enhancing patient safety, minimizing the viral reservoir in the population, decreasing hospital admission, and avoiding influenza and COVID-19 co-infection co-infection. Marital status, previous influenza vaccine uptake, knowledge about flu vaccine safety, perceived benefits, and perceived barriers toward flu vaccination were significantly associated with HCWs flu vaccination uptake.

Despite the ministry of public health’s annual influenza vaccine recommendation, our data revealed that only 32.1% of the surveyed HCWs received their influenza vaccine in the previous season (2019-2020). During the season of 2016-2017, Qatar reached a vaccination coverage rate of 77% among HCWs [14]. Similarly, almost 67.6% of HCWs were vaccinated in the 2016 season in Saudi Arabia [15]. Indeed, these high flu vaccination rates could be attributed to the fact that these countries have made vaccines available for free and run immunization campaigns. During this year (2020-2021) that overlaps with the circulation of SARS-COV2, the influenza vaccine uptake has risen to 80.2% among Lebanese HCWs; this is in line with the study conducted in the United States among nurses [16]. A possible explanation of this rise is that the COVID-19 pandemic has impacted the HCWs willingness to be vaccinated against influenza in the current season which has had significant repercussion on the influenza vaccination coverage for the season 2020/21. Another possible explanation is the success of the world health organization (WHO) [17] and the Centers for Disease Control and Prevention (CDC) recommendations [18] to take the annual flu vaccine for HCWs during the current pandemic.

Concerning HCWs awareness about the flu vaccination, findings of the current survey revealed that 77% of HCWs have a good knowledge level. Lack of knowledge was apparent in the domain related to the prioritization of the target groups when a limited supply of vaccines is available. It is worth noting that more than half of surveyed HCWs considered that pregnant women are not among the prioritized groups for flu vaccination. As a result, a substantial number of HCWs will not recommend the uptake of the influenza vaccine during pregnancy. Moreover, 60% of surveyed HCWs didn’t believe that children aged between 6 months and 5 years are among the prioritized target groups for flu vaccination while several studies have demonstrated the necessity of prioritizing flu vaccination for children to reduce community infection [11, 19]. Another important finding in this study was 23.8% of HCWs were not aware about the timeframe of the flu vaccination and the fact that as long as influenza viruses are circulating, the vaccination could be offered. These knowledge gaps highlight the necessity of increasing HCW awareness about influenza target groups and vaccination timeliness.

Concerning attitude toward flu vaccination, finding of our survey revealed that the majority of HCWs (84.1%) had positive attitude toward flu vaccination. Of note, 28.8% thought that developing natural immunity is better than getting the vaccine. Although a flu infection stimulates the immune system more effectively than the vaccination, this comes at a significant cost as the individual could develop complications and die. It is also worth mentioning that flu vaccines often protects against a specific subtype, and their effectiveness changes each year due to the rapidly evolving influenza virus [2]. It is therefore strongly recommended that persons at risk, such as HCWs, pregnant women, and patients with chronic conditions, get the vaccine at least once a year.

Concerning their attitudes toward the Lebanese government, more than half of HCWs declared that the Lebanese society has more vital challenges other than influenza. This could be due to the worst economic and financial crisis ever experienced in our country that came at a time when the country was and continues to grapple with the COVID-19 pandemic.

The majority of HCWs also considered that the influenza vaccine should be provided free of charge and should be funded by the government and more than 40% of them thought that their health care settings should deliver influenza vaccine at their institution. Thus, ensuring its availability at the health facility and providing it for free could increase vaccination coverage among HCWs.

With respect to practice, more than half of the respondents reported the adoption of positive behaviors such as encouraging patients, relatives, and colleagues to get vaccinated against influenza as well as their participation in trainings and workshops related to flu vaccination. However, less than 50% of HCW were currently promoting the importance of getting the flu vaccine. Given that recommendations from trusted HCWs can help improve flu vaccination acceptance and uptake, it is crucial to raise HCWs awareness on the importance to promote annual flu vaccines by addressing patients hesitancy concerns that might appear during medical appointments.

Another interesting finding from the survey is that 83.3% of HCWs ranked the availability of a sufficient quantity of vaccine as the most significant barrier to vaccine access. The potential shortage will arise as a result of the current year’ high demand. The risk of being co-infected as a result of the COVID-19 pandemic has motivated people to seek flu vaccination in order to avoid COVID-19 complications and reduce the burden on the healthcare system. In addition, the development of side effects (23.57%), severe adverse events (31.07%), and vaccine safety (18.21%) were listed as barriers of vaccination. Since the cost of the vaccine is paid out-of-the-pocket in Lebanon, the cost of the vaccine was stated as a barrier. Similar vaccination barriers were reported in studies conducted in Singapore and Saudi Arabia [20–22].

The main benefits mentioned by HCWs’ uptake or willingness to be vaccinated include enhancing patient safety, minimizing the viral reservoir in the population, decreasing hospital admission due to influenza, and avoiding co-infection by influenza and COVID-19 allowing consequently health services to better cope with COVID-19 complications. Whereas, only 39.29% of HCWs consider flu vaccination as a cost-effective intervention. Similar benefits were reported in previous studies [6, 23, 24].

In our study, we investigated different factors than may affect influenza vaccine uptake in the current 2020-2021 flu season—several significant associations were found. Our results revealed that marital status was a significant influenza vaccine uptake social determinant suggesting that unmarried HCWs were less prone to get the flu vaccine compared to their married counterparts. HCWs might be positively influenced to take the flu vaccine by their partners or they might be afraid of transmitting the infection to their family and children. However, HCWs who had been vaccinated in previous seasons were more prone to receive the 2020/21 flu vaccine. A systematic review conducted by the WHO to evaluate barriers to influenza vaccination intention and behavior showed similar results. In addition, a study conducted in Germany, the flu vaccination in the previous season was reported as the strongest predictor of flu vaccination uptake [25]. Indeed, several systematic reviews have repeatedly documented past behavior as a strong predictor of influenza vaccine acceptance [26, 27]. As expected, our results revealed that a good knowledge level was associated with the vaccination uptake suggesting that HCWs with a high knowledge level are more willing to get the vaccine. Same finding was reported in a study conducted in Dubai [28]. Finally, less perceived barriers and positive perceived benefits were associated with a high willingness to vaccinate. Taken together, these findings could help prioritize health promotion programs aimed at increasing vaccination uptake among HCWs.

Some limitations of this study should be acknowledged: First, the possibility of selection bias due to the convenience sampling strategy that was applied to recruit HCWs and the absence of randomization which limits the generalizability of our findings. Secondly, as the collected data was self-reported, information can be prone to recall bias. Thirdly, responses could be influenced by social desirability bias. Finally, this survey was conducted during the earlier phase of the influenza season, therefore, the attitudes and practices reflected merely the information available at that time.

## Conclusion

Flu vaccination uptake has increased among HCWs during the 2021 flu season compared with the previous one. Continuing education focused on the risk of influenza, the benefits of vaccination uptake and addressing common misconceptions, as well as ensuring free of charge, equitable and convenient access to vaccination are important components to increase the flu vaccination uptake among HCWs.

## Data Availability

Data are available from the corresponding authors upon reasonable request.
